# Adherence to a Dietary Approaches to Stop Hypertension (DASH)-style Diet in Relation to Preeclampsia: A Case-Control Study

**DOI:** 10.1038/s41598-020-65912-2

**Published:** 2020-06-03

**Authors:** Yuan Cao, Yanhua Liu, Xianlan Zhao, Dandan Duan, Weifeng Dou, Wenjun Fu, Huanan Chen, Yacong Bo, Yanfang Qiu, Gaiyun Chen, Quanjun Lyu

**Affiliations:** 1 Department of Nutrition and Food Hygiene, College of Public Health, Zhengzhou University, Zhengzhou, 450001 China; 2 Department of Nutrition, the First Affiliated Hospital of Zhengzhou University, Zhengzhou, 450052 China; 3 Department of Obstetrics, the First Affiliated Hospital of Zhengzhou University, Zhengzhou, 450052 China; 4 Department of Clinical Nutrition, Luoyang New Area People’s Hospital, Luoyang, 471023 China; 5 Jockey Club School of Public Health and Primary Care, the Chinese University of Hong Kong, Hong Kong, China

**Keywords:** Endocrine system and metabolic diseases, Nutrition disorders, Reproductive disorders

## Abstract

Maternal diet is an important potential factor associated with the risk of preeclampsia. However, it is unclear whether adherence to a Dietary Approaches to Stop Hypertension (DASH)-style diet can reduce the development of preeclampsia. To examine the potential association, we conducted a hospital-based case-control study at the First Affiliated Hospital of Zhengzhou University, China. A total of 449 cases with preeclampsia and 449 controls were studied. Dietary information was collected using a validated food frequency questionnaire (FFQ). DASH scores were calculated according to nutrients/food emphasised or minimised in the DASH diet. The calculated DASH scores ranged from 11 to 38 for all of the participants, and the DASH scores of the cases were significantly lower than those of the controls (23.48 ± 4.58 vs 24.51 ± 4.51; *p *= 0.001). Participants in the fourth quartile of the DASH score were 45% less likely to have preeclampsia than those in the first quartile in the crude model (Q4 vs Q1, odds ratio [OR]: 0.55; 95% confidence interval [*CI*]: 0.38, 0.80; *p*_trend_ = 0.001). The relationship remained significant in the model adjusted for multiple confounders, especially for major risk factors of preeclampsia (OR: 0.53; 95% *CI*: 0.36, 0.78; *p*_trend_ = 0.001). Our findings suggest an inverse relationship between adherence to a DASH-style diet and the odds of preeclampsia. Further larger-scale cohort studies or randomised controlled trials are warranted to confirm these relationships.

## Introduction

Preeclampsia is a pregnancy-specific complication that occurs after 20 weeks of gestation and is characterised by hypertension, proteinuria and oedema, with or without multiple organ damage^1^. It is a major cause of foetal and maternal morbidity and mortality worldwide, and affects 2–8% of all pregnancies^2^. Currently, the only definitive treatment for preeclampsia is to terminate the pregnancy^3^. Therefore, primary prevention of preeclampsia is vital.

Evidence indicates that maternal diet is an important potential factor affecting the incidence of preeclampsia^4,5^. Studies have shown that diets rich in vegetables and fruits or various nutrients such as vitamin D, calcium and zinc are associated with lower levels of oxidative stress and inflammation^6,7^, which are both recognised pathogenic factors for preeclampsia^8^. However, individual foods or nutrients are consumed in various characteristic combinations, so nutritional epidemiologists have recommended using overall dietary patterns to find links between diet and diseases^9^. Recent studies have used factor analysis to explore the relationship between dietary patterns and the risk of preeclampsia^10–12^. However, the dietary patterns obtained in these studies were only appropriate for local residents and the results were not comparable. Universal dietary recommendations for the prevention of preeclampsia remain limited.

The Dietary Approaches to Stop Hypertension (DASH) diet is an established dietary pattern rich in whole grains, fruits, vegetables, low-fat dairy and plant proteins from nuts and legumes, but low in red/processed meat, sweets and sugar-sweetened beverages^13^. The DASH diet was originally designed and evaluated for lowering blood pressure^14^, but recent studies have shown its benefits for glioma^15^, nonalcoholic fatty liver disease^16^ and other metabolic disorders such as cardiovascular disease^17^, type 2 diabetes^18^ and gestational diabetes mellitus (GDM)^19^. However, so far, no study has assessed the association between the DASH diet and the odds of preeclampsia.

Although preeclampsia is also characterised by hypertension, its mechanism is not quite the same as that of adult hypertension. Therefore, the aim of this study was to explore the association between adherence to a DASH-style diet and the odds of preeclampsia among pregnant women in China. In addition, considering that the DASH diet was previously found to be associated with a decreased risk of GDM^19^, we performed a sensitivity analysis to exclude the influence of GDM on the results.

## Results

### Participants

Of the 708 inpatients with preeclampsia seen in the hospital, 162 (22.9%) did not meet the inclusion criteria for this study. Of the 546 eligible subjects, 34 (6.2%) cases were excluded due to incomplete surveys or extreme energy intakes (less than 800 or more than 4200 kcal/d)^19^, and 63 (11.5%) cases refused to participate. In the end, 449 cases were retained in the analysis, including 389 (86.7%) cases without GDM and 60 (13.3%) cases with GDM. In addition, 449 eligible controls from the same hospital, matched for age, gestation week and GDM, were enrolled.

### Basic characteristics and dietary intakes of case and control group

The basic characteristics and dietary intakes of the participants according to case/control status are presented in Table 1. There was no significant difference between the two groups in mean age and gestational age. Mean pre-pregnancy BMI was significantly higher in patients with preeclampsia than in the control group (*p* < 0.001). Compared with the control group, the cases with preeclampsia had lower total dietary energy and protein intake, consumed significantly less fish, eggs, fruits, vegetables, nuts and legumes, dairy products and red/processed meat. The preeclampsia cases consumed significantly more sodium and were more likely to be nulliparous than the controls. The calculated DASH scores for all participants ranged from 11 to 38, and the cases had significantly lower adherence to the DASH diet than the controls (23.48 ± 4.58 vs 24.51 ± 4.51; *p* = 0.001).

**Table 1:** General characteristics and dietary intakes of participants based on patients with preeclampsia and control group^a^.

	Cases (n = 449)	Controls (n = 449)	*p*^b^
Age (years)	30.77 ± 4.94	30.98 ± 4.82	0.525
Gestational age (weeks)	34.12 ± 2.88	34.48 ± 2.85	0.063
Pre-pregnancy BMI (kg/m^2^)	23.66 ± 3.87	22.47 ± 3.43	**<0.001**
GDM (%)	60 (13.4)	60 (13.4)	1
Nulliparity (%)	255 (56.8)	299 (66.7)	**0.002**
Physical activity (MET (hour/day))	26.94 ± 3.99	26.44 ± 4.45	0.078
Total energy (kcal/d)	1849.2 ± 502.72	1983.06 ± 529.49	**<0.001**
Carbohydrate (% of total energy)	52.95 ± 7.33	52.32 ± 7.06	0.195
Protein (% of total energy)	13.30 ± 2.37	14.19 ± 2.43	**<0.001**
Fat (% of total energy)	35.14 ± 7.08	34.98 ± 6.19	0.713
Fish (g/d)	4.07 (0.95, 11.37)	7.60 (1.90, 16.29)	**<0.001**
Eggs (g/d)	52.80 (22.63, 79.20)	52.80 (30.17, 79.20)	**0.001**
Whole grains and mixed beans (g/d)	6.91 (1.67, 16.48)	7.14 (1.67, 17.51)	0.361
Fruits (g/d)	281.71 (173.79, 401.57)	335.96 (226.8, 502.19)	**<0.001**
Vegetables (g/d)	299.40 (229.33, 413.39)	335.73 (261.65, 460.67)	**<0.001**
Nuts and legumes (g/d)	21.50 (9.50, 38.41)	25.08 (12.46, 46.01)	**0.003**
Dairy products (g/d)	147.62 (53.57, 262.13)	184.52 (83.33, 280.57)	**0.014**
Red/processed meat (g/d)	27.90 (10.30, 50.35)	42.09 (21.43, 74.58)	**<0.001**
Soft drinks and sweets (g/d)	13.33 (6.67, 30.00)	11.43 (5.33, 26.67)	0.076
Sodium (mg/day)	4761.34 (4133.65, 5445.13)	4555.85 (3976.99, 5135.09)	**<0.001**
DASH score	23.48 ± 4.58	24.51 ± 4.51	**0.001**

BMI: body mass index.

^a^Data are mean ± standard deviation or number (%) or M (*p*_25_, *p*_75_).

^b^Independent sample *t*-test (when satisfying a normal distribution) or Mann-Whitney U test (when not satisfying a normal distribution) for quantitative variables and chi-square test for qualitative variables.

### General characteristics and dietary intakes of the participants by quartiles of DASH score

The general characteristics and dietary intakes of the participants in increasing quartiles of the DASH score are shown in Table 2. The participants in the highest quartiles of the DASH score had higher age and physical activity levels and lower pre-pregnancy BMI than the other quartiles. Adherence to the DASH diet was associated with greater intakes of protein, fish, fruits, vegetables, nuts and legumes, dairy products and red/processed meats. Individuals in the top quartile of the DASH diet had lower intakes of soft drinks and sweets and sodium than those in the bottom quartile. There were no significant differences in consumption of eggs or whole grains and mixed beans between quartiles of the DASH score.

**Table 2:** Basic characteristics and dietary intakes of participants by quartiles of Dietary Approach to Stop Hypertension (DASH) score.

	Quartiles of DASH score^a^				*p*_trend_
	Q1_(lowest)11–20_ (n = 203)	Q2_21–23_ (n = 193)	Q3_24–26_ (n = 235)	Q4_(highest)27–38_ (n = 267)	
Cases (n, %)	116 (57.14)	102 (52.85)	118 (50.21)	113 (42.32)	**0.011**
Age (years)^b^	30.25 ± 4.72	30.32 ± 4.47	30.59 ± 4.82	32.00 ± 5.16	**<0.001**
Gestational age (weeks)^b^	34.66 ± 2.70	34.05 ± 2.85	34.11 ± 2.92	34.37 ± 2.95	0.408
Pre-pregnancy BMI (kg/m^2^)^b^	23.39 ± 3.82	23.41 ± 3.89	22.85 ± 3.57	22.75 ± 3.56	**0.029**
Physical activity (MET (hour/day))^b^	26.55 ± 3.83	26.22 ± 4.30	26.99 ± 4.35	26.88 ± 4.35	**0.201**
Total energy (kcal/d)^b^	2003.99 ± 535.77	1811.50 ± 473.83	1816.03 ± 492.01	2013.07 ± 536.51	0.538
Carbohydrate (% of total energy)^b^	52.53 ± 7.86	52.56 ± 7.32	52.29 ± 6.82	53.06 ± 6.92	0.463
Protein (% of total energy)^b^	13.03 ± 2.07	13.35 ± 2.36	14.05 ± 2.52	14.30 ± 2.52	**<0.001**
Fat (% of total energy)^b^	35.62 ± 7.52	35.44 ± 6.97	35.11 ± 6.13	34.32 ± 6.09	**0.026**
Fish (g/d)^c^	8.72 ± 1.57	12.75 ± 1.61	12.15 ± 1.46	14.78 ± 1.37	**0.035**
Eggs (g/d)^c^	51.86 ± 2.96	54.21 ± 3.04	60.15 ± 2.75	58.43 ± 2.58	0.147
Whole grains and mixed beans (g/d) ^c^	6.40 ± 1.50	8.42 ± 1.54	17.23 ± 1.39	21.55 ± 1.31	0.081
Fruits (g/d) ^c^	250.69 ± 15.91	349.92 ± 16.33	389.83 ± 14.81	452.17 ± 13.90	**<0.001**
Vegetables (g/d) ^c^	280.28 ± 11.18	317.22 ± 11.48	363.65 ± 10.41	442.72 ± 9.77	**<0.001**
Nuts and legumes (g/d)^c^	17.33 ± 1.85	28.37 ± 1.90	34.11 ± 1.73	42.27 ± 1.62	**<0.001**
Dairy products (g/d)^c^	113.59 ± 12.56	191.63 ± 12.89	221.75 ± 11.69	273.94 ± 10.97	**<0.001**
Red/processed meat (g/d)^c^	61.62 ± 2.78	46.74 ± 2.86	45.42 ± 2.59	33.38 ± 2.43	**<0.001**
Soft drinks and sweets (g/d)^c^	27.06 ± 4.08	13.91 ± 4.19	12.61 ± 3.80	8.35 ± 3.57	**0.005**
Sodium (mg/d)^c^	5140.88 ± 44.14	4903.33 ± 45.32	4660.01 ± 41.10	4279.80 ± 38.57	**<0.001**

MET: metabolic equivalent task; DASH: Dietary Approaches to Stop Hypertension; BMI: body mass index; Q, quartile.

^a^Total DASH scores were divided into four ascending categories on an ordinal scale.

^b^Data are presented as mean ± SD; comparisons were made using ANOVA; *p*_trend_ were obtained using linear regression model.

^c^Data are presented as mean ± SEM; comparisons were made using ANCOVA; dietary intakes were adjusted for total energy intake; *p*_trend_ were obtained by considering the median score in each category as a continuous variable.

### Association between adherence to a DASH-style diet and the odds of preeclampsia

The multivariate adjusted odds ratios (ORs) for preeclampsia across the quartiles of the DASH diet score are shown in Table 3. In the crude model, participants in the top quartile of the DASH diet score were 45% less likely to have preeclampsia (Q4 vs Q1, OR: 0.55; 95% confidence interval [*CI*]: 0.38, 0.80; *p*_trend_ = 0.001). After controlling for age, gestational age and GDM, adherence to the DASH diet was still inversely associated with the odds of preeclampsia (Q4 vs Q1, OR: 0.54; 95% *CI*: 0.37, 0.79; *p*_trend_ = 0.001) (Model 1). Further adjustment for total energy intake, physical activity and nulliparity strengthened the association (Q4 vs Q1, OR: 0.49; 95% *CI*: 0.34, 0.72; *p*_trend_ < 0.001) (Model 2). Additional controlling for pre-pregnancy BMI did not influence the association significantly (Q4 vs Q1, OR: 0.53; 95% *CI*: 0.36, 0.78; *p*_trend_ = 0.001) (Model 3).

**Table 3:** Multivariate-adjusted odds ratio for preeclampsia across quartiles of DASH diet scor^a^.

	Quartiles of DASH score				*p*_trend_^b^
	Q1_(lowest)_ (n = 203)	Q2 (n = 193)	Q3 (n = 235)	Q4_(highest)_ (n = 267)	
Crude	1.00	0.84 (0.57, 1.25)	0.76 (0.52, 1.10)	0.55 (0.38, 0.80)	**0.001**
Model 1^c^	1.00	0.82 (0.55, 1.22)	0.74 (0.50, 1.08)	0.54 (0.37, 0.79)	**0.001**
Model 2^d^	1.00	0.72 (0.48, 1.09)	0.61 (0.41, 0.91)	0.49 (0.34, 0.72)	**<0.001**
Model 3^e^	1.00	0.71 (0.47, 1.08)	0.64 (0.43, 0.95)	0.53 (0.36, 0.78)	**0.001**

DASH: Dietary Approaches to Stop Hypertension; BMI: body mass index; Q: quartile.

^a^Binary logistic regression was used to obtain OR and 95% *CI* across increasing quartiles.

^b^Obtained by considering the median score in each category as a continuous variable.

^c^Adjusted for age, gestational age and GDM (yes/no).

^d^Further controlled for energy intake, physical activity and nulliparity (yes/no).

^e^Additionally adjusted for pre-pregnancy BMI.

### Sensitivity analysis

To test the stability of the relationship between adherence to a DASH-style diet and the odds of preeclampsia, we conducted a sensitivity analysis excluding participants with GDM to eliminate the influence of pregnant women with GDM on the results (Table 4). Among 389 pairs of subjects who did not have GDM, participants in the top quartile of the DASH diet score were 39% less likely to have preeclampsia in the crude model (Q4 vs Q1: OR: 0.61; 95% *CI*: 0.41, 0.90; *p*_trend_ = 0.016). In the same way, we adjusted for confounders using the three models mentioned above (except for the confounder of GDM (yes/no)), and found that adherence to the DASH diet was still inversely associated with the odds of preeclampsia.

**Table 4:** Multivariate-adjusted odds ratio for preeclampsia across quartiles of DASH diet score among participants without GDM^a^.

	Quartiles of DASH score				*p*_trend_^b^
	Q1_(lowest)_	Q2	Q3	Q4_(highest)_	
**Without GDM**					
N (case/control)	101/78	87/85	103/101	98/125	
Crude	1	0.79 (0.52, 1.20)	0.79 (0.53, 1.18)	0.61 (0.41, 0.90)	**0.016**
Model 1^c^	1	0.77 (0.50, 1.17)	0.77 (0.51, 1.15)	0.60 (0.40, 0.89)	**0.014**
Model 2^d^	1	0.67 (0.44, 1.04)	0.62 (0.41, 0.94)	0.54 (0.36, 0.82)	**0.004**
Model 3^e^	1	0.67 (0.43, 1.04)	0.62 (0.41, 0.96)	0.58 (0.38, 0.88)	**0.013**

DASH: Dietary Approaches to Stop Hypertension; BMI: body mass index; GDM: Gestational diabetes mellitus; Q: quartile.

^a^Binary logistic regression was used to obtain OR and 95% *CI* across increasing quartiles.

^b^Obtained by considering the median score in each category as a continuous variable.

^c^Adjusted for age and gestational age.

^d^Further controlled for energy intake, physical activity and nulliparity (yes/no).

^e^Additionally adjusted for pre-pregnancy BMI.

## Discussion

This case-control study demonstrated an inverse association between adherence to a DASH-style diet and the odds of preeclampsia. This significant inverse relationship remained in the model adjusted for multiple confounders, especially for major risk factors of preeclampsia including pre-pregnancy BMI and nulliparity. To the best of our knowledge, this is the first case-control study to explore the association between adherence to a DASH-style diet and the odds of preeclampsia in a Chinese population.

Recent studies have assessed the effects of maternal dietary patterns on the risk of preeclampsia^10,12,20^. In a prospective longitudinal cohort study in Norway^21^, four dietary patterns were obtained by factor analysis, of which two were associated with preeclampsia. The dietary pattern characterised by high consumption of vegetables, plant foods and vegetable oils was inversely associated with the odds of developing preeclampsia, while another pattern characterised by processed meat, salty snacks and sweet drinks had the opposite effect^10^. The above results were consistent with our study in terms of the positive scoring and reverse scoring components of a DASH-style diet.

Furthermore, several previous studies have shown that the components of the DASH diet have different effects on preeclampsia^11,21–23^. A meta-analysis reported that a healthy dietary pattern, rich in vegetables, fruits, legumes and whole grains, was significantly associated with lower odds of preeclampsia^11^. These components  had a large overlap with the characteristics of the DASH diet. Whole grains, as an important positive scoring component of the DASH diet, have been linked to reduced preeclampsia and its risk factors in previous studies, attributed to their nutrients and fibre composition. An observational study of 1538 women in the US found that higher total fibre intake reduced the preeclampsia risk, possibly by attenuating pregnancy-associated dyslipidaemia^22^. In addition, a DASH-style diet is high in vegetables and fruits, which are rich in natural antioxidants that have been linked to the recognised pathogenic factor of oxidative stress in preeclampsia^21^. A prospective cohort study in Norway suggested that foods with a high content of added sugar and sugar-sweetened beverages were significantly associated with increased risk of preeclampsia, while foods with natural sugars, such as fresh and dried fruits, were associated with decreased risk of preeclampsia^23^. A possible explanation is that added sugar provides excess energy intake while foods with a high content of natural sugars are rich in dietary fibre. Red or processed meat and sodium are reverse scoring components of the DASH diet. However, the relationship between these components and preeclampsia and its mechanism has not been reported. More research is needed in the future to uncover the specific mechanism of each component of the DASH diet and the synergistic effects among them.

The Chinese diet is characterised by high consumption of plant foods (grains, vegetables, etc.), lack of high-quality proteins (lean meat, fish, dairy products, etc.) and high oil and salt. The characteristics of high sodium, low calcium and low high-quality protein are the opposite of what the DASH diet recommends and may be important factors in the pathogenesis of preeclampsia. Therefore, for Chinese pregnant women, greater adherence to a DASH-style diet may be associated with decreased odds of preeclampsia. Further studies are also needed in other countries where the diets and foods are naturally different.

This study has many advantages. First, it is the first study to assess the association between adherence to a DASH-style diet and the odds of preeclampsia. Second, our study used a matched case-control design to control for the three major confounders, age, gestational age and GDM. In addition, the relatively large sample size of participants improved the validity, and the sensitivity analysis improved the reliability of the results.

However, some limitations must also be considered when interpreting our findings. First, although we used a validated FFQ to measure dietary intake, there were still inevitable measurement errors and recall bias. Nonetheless, the measurement accuracy benefitted from the employment of trained interviewers who used photographs of food portion sizes to administer the FFQ through face-to-face interviews. Second, considering that patients with preeclampsia have a relatively short time from onset to delivery, we only collected information on the dietary intake of the pregnant women during the final three months before delivery to reduce the recall bias of the FFQ, which may have led to a failure to account for the potential influence of early pregnancy diet on preeclampsia.

In conclusion, our findings suggest an inverse relationship between adherence to a DASH-style diet and the odds of preeclampsia. Further larger-scale cohort studies or randomised controlled trials are needed to confirm the relationship.

## Methods

### Participants

This hospital-based case-control study was conducted between March 2016 and June 2019 at the First Affiliated Hospital of Zhengzhou University (located in Zhengzhou, a medium-sized city in central China that has been well developed in recent years). Pregnant women of reproductive age (≥18 years) after the 28^th^ week of pregnancy and with single pregnancy were included. The preeclampsia cases were pregnant women who had been diagnosed with preeclampsia by a physician, according to China’s ‘Diagnosis and treatment guideline of hypertensive disorders in pregnancy’ (2015). In this guideline, preeclampsia is defined as systolic blood pressure (SBP) ≥ 140 mmHg or diastolic blood pressure (DBP) ≥ 90 mmHg after 20 weeks of gestation and accompanied by any of the following characteristics: (1) urinary protein ≥ 0.3 g/24 h, or urinary protein/creatinine ratio ≥ 0.3, or random urinary protein ≥ (+) (the test method used when urinary protein cannot be quantified); (2) no proteinuria but accompanied by damage to any of the following organs or systems: heart, lung, liver, kidney and other important organs, or abnormal changes in the blood system, digestive system, nervous system, placental foetus involved, etc. The controls were enrolled among pregnant women from the same hospital without hypertension or proteinuria and were matched with the case group based on age, gestational weeks and GDM.

The exclusion criteria for subjects in both groups were as follows: (1) patients with heart disease, malignancy, hyperthyroidism, immune system diseases, chronic renal insufficiency, and other endocrine system diseases; and (2) patients with epilepsy, depression and other mental or cognitive dysfunction.

### Calculation of sample size

On the basis of earlier evidence, we assumed that approximately 30% of the people in the control group would have higher DASH scores, and the estimated OR between the higher DASH scores and preeclampsia odds was 0.60^12^. With 90% power (*β* = 0.10), type I error of 0.05 (α = 0.05) and desired *CI* of 0.95, the minimum required sample size in each group was calculated to be 394, that is, a total of 788 participants. Finally, 449 cases and 449 controls were enrolled in this study.

### Assessment of dietary intake

The dietary intake of the study participants during the last three months before delivery was assessed through a valid and reliable semiquantitative food frequency questionnaire (FFQ)^24^, which included 78 items of foods commonly consumed by Chinese. For each food item, 4 possible frequencies (never, every month, every week and every day) and the amount of consumption each time in grams or millilitres were available. The daily intakes of energy (kcal/d) and key nutrients from each food were calculated according to the Chinese Food Composition Tables^25^.

### Assessment of adherence to a dietary approaches to stop hypertension-style diet

A DASH score was calculated based on the FFQ for each participant. According to Fung’s method^26^, the DASH score was constructed based on the foods and nutrients emphasised or minimised in the DASH diet, focusing on eight components: high intake of whole grains; fruits; vegetables; nuts and legumes; and low-fat dairy products, and low intake of red/processed meats; sweetened beverages; and sodium^26^. Because we could not assess the dietary intake of whole grains and low-fat dairy products accurately, we instead calculated whole grains and mixed beans, and dairy products, respectively. Considering that pregnant women consume few sugary drinks, we added sweets to this component.

The scoring criteria and food composition of the DASH-style diet in our study are shown in Table 5. First, the energy-adjusted amounts of components of the DASH-style diet were obtained through the residual method^27^. Subsequently, quintile cut-off points for each component of the DASH-style diet were obtained in all participants. For the five groups of whole grains and mixed beans, fruits, vegetables, nuts and legumes, and dairy products, those in the highest quintile were given 5 points and those in the lowest quintile received 1 point. In contrast, for the remaining three components, the lowest quintile was given 5 points and the highest received 1 point. An overall DASH score ranging from 8 to 40 was obtained by summing the eight component scores. Individuals with higher DASH scores were more likely to follow the DASH diet.

**Table 5:** Scoring criteria and food composition for the DASH-style diet in this case-control study^a^.

Component	Foods	Scoring criteria^a^
Whole grains and mixed beans	Brown rice, wholemeal bread, whole grain cereal, oats, popcorn; Red beans, mung beans and other mixed beans	Positive scoring Q1 = 1 point Q2 = 2 points Q3 = 3 points Q4 = 4 points Q5 = 5 points
Fruits	All fruits	
Vegetables	All vegetables except potatoes and legumes	
Nuts and legumes	Nuts and peanut butter, soybean, tofu, soybean milk, peas	
Dairy	Milk, yoghurt, cottage cheese	
Red and processed meats	Pork, beef, lamb, deli meats, organ meats, hot dogs, sausage, bacon	Reverse scoring: Q1 = 5 points Q2 = 4 points Q3 = 3 points Q4 = 4 points Q5 = 1 point
Beverages and sweets	Carbonated and noncarbonated sweetened beverages, cake, egg tart, pumpkin cake and other desserts	
Sodium	Sum of sodium content of all foods in FFQ (including salt)	

DASH: Dietary Approaches to Stop Hypertension; FFQ: food frequency questionnaire; Q: quintile.

^a^Food intake across Q1 (Low Consumption) to Q5 (High Consumption).

### Assessment of other variables

Information on sociodemographic characteristics, lifestyle habits, pre-pregnancy weight, history of disease, physical activity, menstrual and pregnancy history was obtained through a structured questionnaire. The height and weight of the participants were measured to the nearest 0.1 cm and 0.1 kg, respectively, without shoes and in light clothing. After a rest of ≥10 min, blood pressure was measured at least twice on the left arm with a sphygmomanometer; a third measurement was taken on any occasion when the first two DBP or SBP values differed by more than 3 mmHg or 4 mmHg. At least two measurements were averaged for further analysis. The participants’ physical activity during pregnancy included daily occupational, leisure-time and household-chores, and was expressed as metabolic equivalents (MET) hour/day^28^. Each individual’s body mass index (BMI) was calculated as weight (kg)/height (m^2^).

### Statistical analysis

The Shapiro-Wilk test and Q-Q plots were used to check the normality of the general characteristics and dietary intakes. The general characteristics of the cases and controls were compared using the chi-square test for categorical variables and independent samples *t*-test for continuous variables and were described by number and mean ± SD (normal distribution). Considering that the dietary intakes did not show a normal distribution, we used the Mann-Whitney U test to compare the dietary intakes among cases and controls and described the results using M (*p*_25_–*p*_75_). To assess the association of a DASH-style diet with preeclampsia, the total DASH score was divided into four ascending categories on an ordinal scale. The general characteristics were also compared among quartiles of the DASH score using the chi-square test for categorical variables and one-way ANOVA for continuous variables. The dietary intakes of the participants by quartiles of the DASH score were analysed using analysis of covariance (ANCOVA) adjusted for total energy intake.

The association of the DASH diet with preeclampsia was assessed using binary logistic regression. In addition to the original model, three different models were developed: Model 1 was adjusted for age, gestational age and GDM (yes/no). Model 2 was further adjusted for total energy intake (kcal/d), physical activity (MET) and nulliparity (yes/no). Model 3 comprised Model 2 plus an adjustment for pre-pregnancy BMI. In all models, the first quartile of the DASH score were considered as the reference group. The overall trend of OR across increasing quartiles of the DASH score was examined by considering the median score in each category as a continuous variable^15^. All probability values below 0.05 were considered statistically significant. All of the statistical analyses were performed using SPSS (version 25.0; SPSS Inc., Chicago, IL).

### Ethical approval

This study was approved by the Ethics Committee of Scientific Research and Clinical Trials of the First Affiliated Hospital of Zhengzhou University and written informed consent was obtained from all participants. All of the procedures were performed in accordance with the approved guidelines laid down in the Declaration of Helsinki.

## Data Availability

Additional data are available from the corresponding author upon reasonable request.
